# The Georgia Pasteur Institute and Laboratory

**Published:** 1900-12

**Authors:** 


### THE GEORGIA PASTEUR INSTITUTE AND LABORATORY.

THE charter for this institution was signed September 8th, and sixty days later it became a reality. November 10th, it opened its doors thoroughly equipped to treat patients bitten by rabid animals, and to make pathological and bacteriological examinations for the profession.

This institution is the result of a paper read before the Medical Association of Georgia at their meeting here in April. In this paper Dr. Slack showed the necessity for such an institution in the south. The association indorsed the establishment of a Pasteur Institute and Laboratory in Atlanta, and on motion of its late beloved and venerable ex-president, Dr. A. W. Griggs, appointed a committee with power to act. This committee has been working hard ever since, and as the result of their labors the institute is now open at 34 Auburn avenue.

As to the necessity for such an institution in the south there can be no doubt, for there have been six deaths from hydrophobia of white persons since April in Georgia. On five of these that old fetish, the relic of a superstitious age, the mad stone was used.

As to the value of the Pasteur treatment in preventing the development of rabies, it is as well established as vaccination against smallpox and antitoxin in the treatment of diphtheria. The mortality has been reduced to less than one-half of one per cent. A thoroughly equipped pathological and bacteriological laboratory, where the profession can have such investigations promptly and accurately made, will be a great help to scientific medicine, and we wish the institute much success.

